# Novel Biomarkers of Bone Metabolism

**DOI:** 10.3390/nu16050605

**Published:** 2024-02-22

**Authors:** Sara Fernández-Villabrille, Beatriz Martín-Carro, Julia Martín-Vírgala, Mª del Mar Rodríguez-Santamaria, Francisco Baena-Huerta, Juan Rafael Muñoz-Castañeda, José Luis Fernández-Martín, Cristina Alonso-Montes, Manuel Naves-Díaz, Natalia Carrillo-López, Sara Panizo

**Affiliations:** 1 Instituto de Investigación Sanitaria del Principado de Asturias (ISPA), 33011 Oviedo, Spain; 2 Redes de Investigación Cooperativa Orientadas a Resultados en Salud (RICORS), RICORS2040 (Kidney Disease), 28040 Madrid, Spain; 3 Bone and Mineral Research Unit, Hospital Universitario Central de Asturias, 33011 Oviedo, Spain; 4 Nephrology Service, Reina Sofia University Hospital, Maimonides Biomedical Research Institute of Cordoba (IMIBIC), University of Córdoba, 14004 Córdoba, Spain

**Keywords:** bone mineral density, vascular calcification, biomarker, chronic kidney disease

## Abstract

Bone represents a metabolically active tissue subject to continuous remodeling orchestrated by the dynamic interplay between osteoblasts and osteoclasts. These cellular processes are modulated by a complex interplay of biochemical and mechanical factors, which are instrumental in assessing bone remodeling. This comprehensive evaluation aids in detecting disorders arising from imbalances between bone formation and reabsorption. Osteoporosis, characterized by a reduction in bone mass and strength leading to heightened bone fragility and susceptibility to fractures, is one of the more prevalent chronic diseases. Some epidemiological studies, especially in patients with chronic kidney disease (CKD), have identified an association between osteoporosis and vascular calcification. Notably, low bone mineral density has been linked to an increased incidence of aortic calcification, with shared molecules, mechanisms, and pathways between the two processes. Certain molecules emerging from these shared pathways can serve as biomarkers for bone and mineral metabolism. Detecting and evaluating these alterations early is crucial, requiring the identification of biomarkers that are reliable for early intervention. While traditional biomarkers for bone remodeling and vascular calcification exist, they suffer from limitations such as low specificity, low sensitivity, and conflicting results across studies. In response, efforts are underway to explore new, more specific biomarkers that can detect alterations at earlier stages. The aim of this review is to comprehensively examine some of the emerging biomarkers in mineral metabolism and their correlation with bone mineral density, fracture risk, and vascular calcification as well as their potential use in clinical practice.

## 1. Introduction

Bone constitutes a mineralized porous structure composed predominantly of organic and inorganic compounds. The organic component primarily consists of a mineralized extracellular matrix formed by collagenous fibers and non-collagenous proteins, along with cells such as osteoblasts, osteoclasts, and osteocytes (osteoblasts entrapped within the matrix) [1,2]. The principal functions of bones encompass providing structural support and protection to internal organs while serving as anchorage points for the musculature of the locomotor system. Additionally, bones play a vital metabolic role by functioning as reservoirs for essential minerals such as calcium (Ca) and phosphorus (P), crucial for maintaining mineral homeostasis. The inorganic component of bone constitutes approximately 60% of its dry mass. Bones comprise essential minerals, including calcium, phosphorus, and magnesium, which bind together to facilitate the deposition of hydroxyapatite crystals. This process significantly contributes to bone mineralization [3].

In recent years, the skeleton has been recognized as an endocrine organ with the capacity to synthesize various substances such as Fibroblast Growth Factor 23 (FGF-23), sclerostin, and osteocalcin [4,5]. Beyond its role in regulating its own metabolism, the skeletal system also influences other physiological processes, including vascular calcification, glucose metabolism, and left ventricular hypertrophy [6,7].

Bone, as a metabolically active tissue, undergoes continuous remodeling characterized by the removal of old bone through osteoclasts and the subsequent replacement with new bone formed by osteoblasts, primarily through the processes of resorption and formation [8]. Various cell types participate in this dynamic remodeling process, regulated by a myriad of biochemical and mechanical components. This intricate interplay allows for the assessment of bone remodeling as a valuable complement to bone mineral density (BMD) measurements, aiding in the detection of disorders resulting from imbalances between formation and resorption.

Bone is a dynamic and living tissue that undergoes substantial transformations throughout its lifespan. An array of factors, encompassing genetic anomalies, hormonal disorders, and nutritional deficiencies, interfere with the development of a strong and healthy skeleton [9,10]. During childhood and adolescence, bone experiences a phase of rapid growth and development, characterized by prevalent bone formation processes, culminating in peak bone density typically around the age of 20–25 [11]. Adequate nutrition, particularly ensuring sufficient intake of calcium, vitamin D, and proteins, is crucial for optimal bone development at this stage [9,12]. Remodeling continues throughout life so that most of the adult skeleton is replaced approximately every 10 years [13] In young adulthood, the balance between bone formation and resorption remains relatively stable. As individuals progress through the aging process, a discernible shift occurs in the balance between bone formation and resorption, tilting towards augmented resorption rates. Consequently, there ensues a gradual decline in BMD, heightening susceptibility to skeletal fragility. Sustaining an optimal body weight, adequate intake of calcium and vitamin D, and regular physical activity remain essential for bone health during this stage [9]. In advanced age, bone loss may accelerate with the consequent increase in the risk of osteoporosis and fragility fractures, underscoring the imperative of proactive measures aimed at preserving skeletal integrity and averting adverse outcomes [10].

One of the diseases characterized by a higher prevalence of bone tissue alterations is chronic kidney disease (CKD) and its associated bone and mineral disorders (CKD-MBD). In this context, disorders in CKD-MBD are prevalent and associated with accelerated aging [14]. CKD induces premature aging of the skeleton, elevating the incidence of fractures even in early stages (CKD stage 2) with a disproportionately higher relative risk at younger ages [15,16,17].

Osteoporosis, characterized by a reduction in bone mass and strength leading to increased bone fragility and fractures, is defined by the World Health Organization as a BMD at the hip and/or spine less than 2.5 standard deviations compared to the bone mass of healthy young adults, determined through dual-energy X-ray absorptiometry (DXA) [18,19]. Its incidence is on the rise with the aging population, affecting over 200 million people worldwide and resulting in approximately 8.9 million osteoporotic fractures annually [20]. Osteoporotic fractures contribute to increased mortality within the first year (20–40%) and loss of independence and quality of life in affected individuals [21]. Additionally, several epidemiological studies suggest an association between bone loss and vascular calcification, with low BMD linked to a higher incidence of aortic calcification [22,23,24,25,26]. Patients with CKD face an increased risk of osteoporotic fractures [17] and an accelerated development of vascular calcification [27].

Suitable markers of bone remodeling should possess characteristics such as simple sample collection, non-invasive determination, specificity to bone metabolism, and a correct correlation with standard techniques in bone remodeling analysis (bone biopsy, histomorphometry, X-rays, and isotopic studies with labeled calcium). Furthermore, they should respond to treatments for diseases affecting bone metabolism.

Bone remodeling markers can be categorized as classical and novel. While this review primarily focuses on novel biomarkers, a summary of classical ones is also provided. Classical markers include biomarkers of bone homeostasis, bone formation, and bone resorption (refer to Table 1). Although widely used in clinical settings, classical biomarkers like calcium (Ca), phosphorus (P), parathyroid hormone (PTH), bone-specific alkaline phosphatase (BALP), osteocalcin, type I procollagen extension peptides (P1NP and P1CP), amino-NTX1- and carboxyl-terminal cross-linked telopeptides (CTX1- for type 1 collagen) have limitations, including low specificity (due to synthesis in tissues other than bone), low sensitivity, susceptibility to dietary, age, gender, or circadian rhythm influences, and potential alteration in the presence of renal failure. Some, such as galactosyl hydroxylysine [28], still have limited clinical application due to the lack of simple routine methods for their determination. Moreover, the contradictory results in different studies evaluating their role as biomarkers of osteoporosis or vascular calcification pose challenges. For instance, consider the impact of osteopontin (OPN), a classical biomarker of bone resorption, on osteoblasts: certain investigations suggest that OPN induces the proliferation and differentiation of osteoblasts and promotes mineralization in bone [29]. In contrast, other studies present conflicting evidence, proposing that OPN inhibits these processes in osteoblasts [30]. There are also studies that report no discernible effect of OPN on osteoblast development [31]. Despite these drawbacks, classical biomarkers remain invaluable in clinical practice for monitoring and analyzing patient progression.

In an effort to address these challenges, researchers are actively seeking novel biomarkers that offer greater specificity. Table 2 delineates the criteria that effective biomarkers of bone metabolism must fulfill.

Given the significant association between bone loss and vascular calcification [32], this review will also examine the correlation of bone biomarkers with cardiovascular risk, specifically focusing on their relationship with vascular calcification.

## 2. Novel Bone Markers

### 2.1. Receptor Activator of Nuclear Factor Kappa B Ligand and Osteoprotegerin

The Receptor Activator of Nuclear Factor kappa B (RANK)/RANK ligand (RANKL)/osteoprotegerin (OPG) system, and the more recently discovered member, leucine-rich repeat-containing G protein-coupled receptor 4 (LGR4), play pivotal roles in both bone and vascular mineralization. While the system’s involvement in bone maintenance is well-established, recent studies have attributed a significant role to this system in vascular smooth muscle cell (VSMC) calcification. In bone, osteoblasts synthesize and secrete RANKL, which binds to its transmembrane receptor RANK in osteoclast precursors. This binding promotes the maturation, activation, and survival of osteoclasts, thereby increasing bone resorption and loss. Simultaneously, osteoblasts secrete OPG, a soluble receptor of RANKL, which prevents the binding of RANKL to RANK, thereby regulating osteoclastogenesis [33]. The LGR4 extracellular domain competes with RANK for binding to RANKL, resulting in the inhibition of osteoclast differentiation and a subsequent reduction in osteoclastic bone resorption. LGR4 is essential for bone formation by promoting the maturation and mineralization of osteoblasts [34]. Recent findings indicate that LGR4 is expressed in mature osteoclasts, where it inhibits osteoclastogenesis through two distinct mechanisms: (1) by preventing the binding of RANKL to RANK and (2) through the binding of RANKL to LGR4, activating the GSK3-β signaling pathway, suppressing the expression of NFATc1, and inhibiting osteoclast differentiation [34,35].

Some factors, including PTH, facilitate osteoclast differentiation by augmenting RANKL production in osteoblasts and concurrently inhibiting OPG synthesis [33]. The expression of this system is additionally modulated by various cytokines and glucocorticoids [25]. Numerous studies have delved into the impact of diet on BMD. Traditionally, the focus has centered on exploring the influence of the Mediterranean diet on classical biomarkers such as calcium and vitamin D. Nevertheless, a distinct study brought to light that beta-carotene and isoflavones, particularly genistein, exhibited a decelerating effect on bone resorption. This effect manifested through a reduction in serum levels of bone resorption markers, such as RANKL, concomitant with an increase in markers indicative of new bone formation, such as OPG [36,37]. Furthermore, various types of dietary fatty acids can also modulate the RANK/RANKL/OPG system, each with distinct osteogenic potential. The authors suggest that olive oil, in particular, may prevent the development and progression of osteoclast-related diseases [38].

The identification that OPG knockout mice develop osteoporosis and severe arterial calcification [39], along with the observation that RANKL expression increases in calcified arterial tissue [40], and evidence that RANKL induces VSMC calcification in vitro, with OPG preventing this process [41,42], suggests that the RANK/RANKL/OPG/LGR4 axis may serve as a crucial autocrine/paracrine system involved in both bone loss and vascular calcification. Consequently, it was anticipated that Denosumab, a human monoclonal antibody against RANKL used for osteoporosis treatment, could prevent or delay the progression of vascular calcification. Indeed, Helas et al. demonstrated that Denosumab reduced vascular calcium deposition in glucocorticoid-induced osteoporosis in mice, affirming the existence of a connection between the bone and vascular systems [43]. However, the FREEDOM study involving osteoporotic patients revealed that the frequency of aortic calcification progression and adverse cardiovascular events was similar between women in the placebo and Denosumab groups, despite the improvement in BMD and reduction in fracture risk due to the treatment [44]. These findings highlight the necessity for studies investigating the regulatory mechanisms of this pathway in both bone and vessels, ensuring that strategies aimed at bone protection do not inadvertently result in counterproductive effects on vascular calcification.

The role of OPG and RANKL as serum biomarkers has been reported by several authors (Table 3). The RANKL/OPG ratio is crucial in determining the degree of bone remodeling and bone mass [40]. Postmenopausal women with low BMD demonstrated lower serum OPG levels and a higher RANKL/OPG ratio compared to women with normal BMD [45]. Similarly, in a study involving rheumatoid arthritis patients, those with osteoporosis exhibited lower serum OPG levels and higher RANKL levels compared to patients with normal BMD [46,47].

Although consensus is lacking regarding their relation with vascular calcifications (Table 3), several studies in CKD patients have associated vascular calcification with elevated OPG serum levels [48,49]. However, in another study involving patients with ischemic coronary disease [50], circulating OPG levels exhibited a negative correlation with total coronary artery calcification, no correlation with serum RANKL concentration, and a positive correlation between the RANKL/OPG ratio and total coronary artery calcification. These variations in results may be attributed to the timing of disease progression during biomarker determinations and the challenge of discerning whether the biomarker is being produced by the bone undergoing mineralization, the vessel undergoing calcification, or a vessel protected from calcification. Another possible cause that explains these differences is the different populations used, which is commonly called selection bias.

A study proposed the feasibility of detecting LGR4 in serum [51], positing that it may sequester RANKL, thereby preventing its binding to RANK in bone. However, the biological significance of this discovery remains undetermined. In an unpublished preliminary study conducted by our group, rats with chronic renal failure, manifesting vascular calcification and bone demineralization, revealed elevated serum RANK/OPG ratios, with no concurrent alterations in the serum levels of LGR4. Further investigations are essential to elucidate the potential role of LGR4 as a serum biomarker for bone loss or vascular calcification.

### 2.2. Sclerostin and Dickkopf1

The Wnt/β-catenin pathway constitutes an intracellular signaling pathway that serves as a primary regulator of bone formation and it is involved in the progression of vascular calcification. Wnt/β-catenin governs osteoblast activity [52]. Endogenous antagonists of the Wnt/β-catenin pathway include, among others, sclerostin (also known as Sost) and dickkopf1 (Dkk1). In bone, PTH acts as a key modulator of this pathway: PTH inhibits sclerostin, thereby enhancing bone formation. Similarly, PTH inhibition leads to increased expression of sclerostin, underlining the close relationship between sclerostin and PTH [53]. However, there is a lack of consensus regarding the effects of PTH on Dkk1 [54,55].

Sclerostin and Dkk1 are predominantly secreted by osteocytes into the circulation, and their serum levels are indicative of inhibited bone formation. Circulating sclerostin levels are influenced by gender, increase with age, and exhibit higher concentrations in elderly subjects compared to younger counterparts with similar BMD [56]. Moreover, exercise downregulates sclerostin [57,58]. Notably, postmenopausal women demonstrate increased sclerostin levels [59]. Although it might be expected that sclerostin and Dkk1 are inversely correlated with BMD in postmenopausal women, several studies have revealeda positive association between BMD and sclerostin and Dkk1 [60,61,62]. For instance, a recent study found a positive correlation between sclerostin and Dkk1 expression in bone from postmenopausal women with osteoporosis and BMD, with their serum levels reflecting their bone levels [60]. This discrepancy may be explained by the fact that bone sclerostin and Dkk1 are primarily produced by live osteocytes, and their levels may reflect osteocyte numbers (associated with higher BMD). The number of live osteocytes typically decreases with age, leading to lower levels of bone sclerostin and Dkk1. Conversely, other studies suggest a negative association between serum sclerostin levels and BMD [63,64], with elevated circulating sclerostin levels identified as a strong and independent risk factor for osteoporosis-related fractures in postmenopausal women [65]. The age-dependent decline in glomerular filtration rate should be considered when interpreting circulating sclerostin levels, necessitating further studies to ascertain its value as a bone biomarker. A recent study suggests that the sclerostin/PTH ratio best defines bone status since the ratio can integrate both PTH-dependent bone formation and a lower rate of bone formation associated with high levels of sclerostin [66].

In the vasculature, the role of sclerostin and Dkk1 remains controversial. Some studies in CKD patients report increased serum sclerostin levels positively correlated with aortic calcification [67]. However, another CKD study indicates that higher sclerostin levels are associated with lower aortic calcification and a better survival rate [64]. In a mouse model of adenine diet-induced vascular calcification, sclerostin knockout mice exhibited more extensive vascular calcification than wild-type mice [68]. Conversely, in another mouse model of vascular calcification induced by warfarin, anti-sclerostin treatment increased aortic and vascular calcification, suggesting a protective role for sclerostin against vascular calcification [68]. These results are in agreement with studies that have shown an association between the presence of vascular calcification in the aorta of rats with chronic renal failure with a decrease in aortic Sost gene expression levels [41]. However, a study in diabetic rats with chronic renal failure demonstrated that monoclonal antibodies against Dkk1 prevented both bone and vascular damage [69]. More research is imperative to resolve these conflicting findings.

Romosozumab, an anti-sclerostin treatment, presents a promising avenue for preventing and treating fractures in osteoporosis among postmenopausal women; however, potential negative cardiovascular effects need careful consideration [41,68,70,71].

### 2.3. Periostin

Periostin, also known as a specific osteoblastic factor, is a non-collagenous protein predominantly expressed in the periosteum, a fibrous membrane covering the bone surface and connected to the muscles. The periosteum consists of two layers, with the outer layer primarily composed of fibroblasts and the inner layer housing bone progenitor cells, osteoblasts, nerves, and blood vessels. Periostin is also expressed in other connective tissues, including the periodontal ligament, muscle fascia, aorta, heart valves, and tendons, contributing to their structural integrity and participating in reparative processes. In bone, periostin regulates collagen crosslinking that contributes to bone strength [72]. Periostin-deficient mice exhibit low BMD, impaired microarchitecture and decreased bone strength [73].

In addition, periostin may serve as a crucial mediator of the effects of PTH on the Wnt/β-catenin pathway. Intermittent PTH administration stimulates periostin and inhibits sclerostin synthesis in bone and osteoblasts in vitro. Moreover, the addition of recombinant periostin also suppresses the expression of sclerostin [73,74]. Teriparatide (PTH 1–34) therapy increases periostin secretion in postmenopausal women [75]. In animal models, hypocaloric diets diminished serum periostin [76].

Periostin is a soluble factor detectable in serum and plasma. Studies examining its role as a biomarker of bone metabolism present conflicting results. The OFELY (Os des Femmes de Lyon) study positively associated serum periostin levels with fracture risk in postmenopausal women [77]. This unexpected finding is explained as an adaptive metabolic response of periosteal cells to maintain bone. Similar results were observed in another study, where plasma periostin levels were higher in postmenopausal women with non-vertebral fractures, suggesting that plasma periostin may be a potential biomarker for osteoporotic fracture risk, particularly in non-spinal skeletal sites [78]. However, another study found no differences in periostin levels between postmenopausal women with normal and low BMD [79].

The impact of periostin on vascular calcification is less explored, although it is considered a promotor of vascular calcifcation. A recent study demonstrated that recombinant periostin in vitro promoted the phenotypic transdifferentiation of VSMCs to a calcifying phenotype. Ex vivo, recombinant periostin accelerated aortic calcification, partly through excessive activation of glycolysis and imbalanced mitochondrial homeostasis [80]. Furthermore, the study observed a positive association between plasma periostin levels and the calcification score (Agatston score) in patients with angina pectoris or suspected coronary artery disease [80]. Another recent study noted increased periostin expression in calcified VSMCs and calcified arteries of diabetic rats. It was further described that periostin acts on calcification by blocking autophagic flow [81]. Additionally, periostin increases human VSMC calcification via activation of β-Catenin, and serum periostin levels were higher in hemodialysis patients compared with healthy controls [82]. Therefore, periostin appears to regulate various processes influencing vascular calcification.

### 2.4. Sphingosine-1-Phosphate

Sphingosine-1-phosphate (S1P) is a lipid mediator that acts through G protein-coupled receptors, controlling various cell functions. In bone, S1P regulates osteoblast survival and migration, while also stimulating RANKL synthesis and promoting osteoclast differentiation [83]. Additionally, S1P controls the dynamic migration of osteoclast precursors between blood and bone through its receptors S1PR1 and S1PR2, which exert opposing actions. S1PR1 mediates positive chemotaxis toward S1P in osteoclast precursors, while S1PR2 directs negative chemotaxis (or chemorepulsion) by generating gradients (Table 4) [84].

Some recent articles show that increased serum levels of S1P are responsible for reduced levels of BMD and increased levels of bone resorption markers, which are associated with an increased risk of osteoporosis [85,86]. In human studies, circulating levels of S1P are increased in postmenopausal women compared to premenopausal women and men. Furthermore, elevated serum levels are associated with lower BMD in postmenopausal and premenopausal women, as well as in men [87]. Additionally, serum levels of S1P are positively correlated with bone resorption markers [87]. Other studies suggest that S1P may be a potential predictor of fracture risk in postmenopausal women [85,86,88].

The effects of S1P in vascular calcification are less well evaluated. A recent study observed that S1P is increased in phosphate-induced VSMC calcification [89], and the exogenous addition of S1P to VSMCs accelerated their calcification [89]. In the interstitial cells of the valve, S1P caused increases in bone morphogenetic protein 2 (BMP2), alkaline phosphatase (ALP), and calcification, as well as proinflammatory effects [90]. Currently, its potential role as a serum biomarker of vascular calcification is still unknown.

### 2.5. microRNAs (miRs)

microRNAs (miRs) are small, single-stranded, non-coding RNAs (18–25 nucleotides) that mediate post-transcriptional gene silencing, regulating various processes in the organism, including organogenesis, cell proliferation, differentiation, and apoptosis. Several studies have identified miRs as key regulators of skeletal-related genes, with potential mediation of cardiovascular complications [91,92,93]. The stability of miRs in plasma, whether free or bound to proteins, makes them potential biomarkers. Moreover, miRs can be easily detected in serum/plasma samples through simple and inexpensive techniques, offering high specificity. Additionally, miRs could play a crucial role in the development of new therapeutic strategies for managing osteoporosis and vascular calcification.

**Figure 1:**
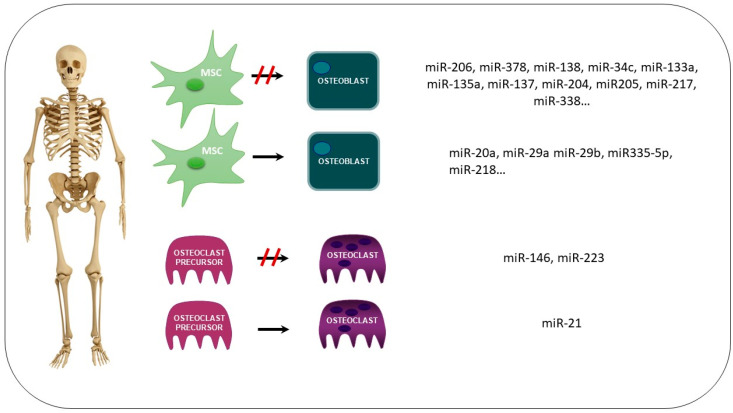
Summary of the role of miRs controlling the differentiation and function of osteoblasts and osteoclasts. miRs regulate the differentiation of mesenchymal stem cells to osteoblasts and osteoblast differentiation (to inhibit or to promote it). miRs can also inhibit or stimulate the differentiation of osteoclasts. Several miRs involved in each process are shown in the box (MSC: mesenchymal stem cells).

miRs play a significant role in controlling the differentiation and function of osteoblasts and osteoclasts (Figure 1). Various positive and negative regulatory miRs, from mesenchymal stem cell differentiation to osteoblasts, have been identified. Numerous miRs inhibit osteoblast differentiation by controlling factors such as connexins, Runx2, and BMPs, including miR-206, miR-378, miR-138, miR-34c, miR-133a, miR-135a, miR-137, miR-204, miR-205, miR-217, and miR-338, among others [94,95,96,97,98]. Equally extensive is the list of miRs promoting osteoblast differentiation, including those controlling sclerostin, Dkk1, various histone deacetylases, or factors degrading Runx2, such as miR-20a, miR-29a, miR-29b, miR335-5p, miR-218, etc. [99,100,101]. Less is known about the regulation of osteoclasts by miRNAs. For example, miR-21 stimulates the differentiation of macrophages to osteoclasts, while miR-223 and miR-146 inhibit the differentiation of osteoclast precursors to osteoclasts [102,103,104].

In the last decade, various studies have identified different miRs as circulating markers of osteoporosis. For instance, miR-133a levels in circulating monocytes (osteoclast precursors) showed a negative association with BMD in postmenopausal women [105]. Studies have found correlations between circulating miRs (miR-21-5p, miR-93-5p, miR-100-5p, and miR-125b-5p) and their levels in bone tissue with BMD in osteoporotic patients showing a significant increase in serum and bone [106]. Another study identified eight miRs (miR-152-3p, miR-30e-5p, miR-140-5p, miR-324-3p, miR-19b-3p, miR-335-5p, miR-19a-3p, and miR-550a-3p) associated with osteoporotic fractures across genders and age groups [107]. Additionally, miR-21 and miR-133a were found to be associated with BMD in patients with osteoporosis and osteopenia [108] and miR-194-5p was suggested as a discriminator between osteoporosis and osteopenia [109]. The list of miRs suggested as biomarkers for an early diagnosis of osteoporosis is extensive in the literature and consensus is needed to indicate which panel of circulating miRs would serve in all populations.

In the first study analyzing an miR-dependent mechanism in the progression of vascular calcification, miR-125b deregulation led to the transition of human coronary artery arterioles into osteoblast-like cells via the osterix transcription factor (Osx). The inhibition of miR-125b promoted alkaline phosphatase activity and matrix mineralization in vitro [91]. It has also been shown that miR-26a and miR-30 regulate Smad1; miR-30, miR-133, miR-204 and miR-211 regulate Runx2; and miR-145 and miR-673 regulate Osx, among others [110], but the list is much more extensive.

Also in recent years, the role of miRs as predictors of vascular calcification has been studied with great interest. Similar to bone, different miRs have been found that indicate the presence of vascular calcification. Studies have identified miR-125 as a biomarker of calcification in the elderly population [111] and in patients with CKD [112]. A recent own study demonstrated that miR-145 and miR-486 regulate VSMC transdifferentiation, loss of the contractile phenotype, and increase in osteogenic genes, preceding VSMC calcification. Both miRs were significantly decreased in the serum of rats and humans with aortic calcification, suggesting their potential as biomarkers for early detection [113]. Similar to the situation in bone, the list of miR biomarkers for vascular calcification is expanding, and studies should be directed toward validating them across diverse populations (Table 5).

While circulating miRs are considered excellent biomarkers due to their measurability, stability, and ease of detection through inexpensive techniques, there are also challenges. Lifestyle and disease can trigger interindividual variations in circulating miR levels, limiting their clinical utility. Factors such as gender, diet, smoking, circadian rhythm, and sports practice can modify the levels of different miRs. Consequently, studies of circulating miRs require strict definition of inclusion and exclusion criteria for patients with these influencing factors [114].

## 3. Conclusions

Bone loss and vascular calcification represent significant public health challenges. They share common mechanisms, including oxidative stress and inflammation, and are influenced by risk factors such as advanced age, CKD, hypertension, and diabetes, among others. Early diagnosis of these alterations would represent an important advance.

However, existing studies have presented contradictory results for many biomarkers. These discrepancies could be attributed to variations in the progression stages of the disease, challenges in distinguishing whether a detected molecule in serum is synthesized by the vessel or the bone, and differences in assays, study populations, and the diverse causes of the disease, among other factors. Nowadays, the biomarkers under investigation primarily serve as supplementary tools to existing diagnostic techniques, including bone histomorphometry, bone biopsy, dual-energy X-ray absorptiometry (DXA), and isotopic studies with labeled calcium for osteoporosis detection or X-ray assessments for vascular calcification scoring. As stated in the present review, new biomarkers of mineral metabolism alterations are being sought. Research should focus on answering if any of them are sensitive and specific enough for future clinical use for the diagnosis and monitoring of patients.

## Figures and Tables

**Table 1:** Classification of biomarkers of bone remodeling.

**BIOMARKERS OF BONE REMODELING**
**Classic Markers**
** Markers of bone homeostasis**
Calcium (Ca)
Phosphorus (P)
Parathyroid hormone (PTH)
1,25(OH)_2_D_3_ (calcitriol)
Fibroblast growth factor 23 (FGF23)
Klotho
** Markers of bone formation**
Total alkaline phosphatase (ALP)
Bone-specific alkaline phosphatase (BALP)
Osteocalcin (*)
Type I procollagen extension peptides (P1NP and P1CP)
** Bone Resorption Markers**
Urinary calcium excretion
Urinary hydroxyproline excretion
Urinary hydroxylysine excretion
Urinary pyridinoline and deoxypyridinoline excretion
Tartrate resistant acid phosphatase (TRACP)
Amino and carboxyl terminal cross-linked telopeptides of type 1 collagen
Cathepsin K (CatK).
Osteopontin (OPN)
**Novel Bone Markers**
Receptor Activator of NFkappa B ligand (RANKL) and osteoprotegerin (OPG)
Sclerostin (Sost) and Dickkopf1 (Dkk1)
Periostin
Sphingosine-1-phosphate (S1P)
MicroRNAs (miRs)

*: bone rate formation marker.

**Table 2:** Characteristics of an ideal bone metabolism biomarker.

Characteristics of a Good Biomarker of Bone Remodeling
Ease of sample collection.
Non-invasive determination.
Specificity to bone metabolism.
Strong correlation with reference techniques in remodeling analysis (bone histomorphometry, bone biopsy, radiographs, and studies involving isotopes with labeled calcium).
Responsiveness to treatments of diseases affecting bone metabolism.

**Table 3:** Examples of studies on the role of OPG and RANKL as biomarkers of low bone mineral density and vascular calcification.

	OPG—RANKL Levels	Subjects	General Results of the Studies
**Bone mass**	Low serum OPG High serum RANKL/OPG	Postmenopausal women (low BMD versus normal BMD) [45].	Consensus
	Low serum OPG High serum RANKL	Rheumatoid arthritis patients (osteoporotic versus normal BMD) [46,47].	
**Vascular** **calcification**	High serum OPG	CKD patients (with vascular calcification versus no vascular calcification) [48,49].	Controversial
	High serum RANKL/OPG	Ischemic coronary disease (with arterial calcification versus no calcification) [50].	

OPG: osteoprotegerin; RANKL; Receptor Activator of NF kappa B ligand; BMD: bone mineral density; CKD: chronic kidney disease.

**Table 4:** Summary of Sphingosine-1-phosphate actions in bone cells.

Sphingosine-1-Phosphate (S1P)	
**Osteoblast**	Survival and migration
	RANKL synthesis
**Osteoclast**	Osteoclast precursors dynamic migration:
	Receptor S1PR1: positive chemotaxis
	Receptor S1PR2: negative chemotaxis (chemorepulsion)

**Table 5:** Most relevant microRNA (miR) promoters or inhibitors of vascular calcification, whose quantification in serum has shown potential as biomarkers for this condition and its corresponding target genes.

	miRNA	Target Gen	Population Studied
**Vascular calcification inhibitors**	miR-16	VEGFA	CKD patients
	miR-17	VEGFA	CKD patients
	miR-106b	VEGFA	CKD patients
	miR-125b	Osterix	Older adults CKD patients
	miR-145	KLF-4, Osterix, α-actin	General population CKD patients
	miR-155	Akt-FOXO3a, AT1R	CKD patients
	miR-211	RUNX2	CKD patients
	miR-378a-3p	CTGF	Older adults CKD patients
	miR-486	RUNX2, Osterix	General population
**Vascular calcification promotors**	miR-29a	DKK1	CKD patients
	miR-29b	ACVR2A, CTNNBIP1	CKD patients
	miR-221	Pit-1	CKD patients
	miR-223	Mef2c, Rhob, NFIA	Obstructive coronary disease patients CKD patients

VEGFA, vascular endothelial growth factor A; KLF4, Krüppel-like factor 4; Akt, protein kinase B; FOXO3a, forkhead box o3a; AT1R, angiotensin receptor 1; RUNX2, Runt-related transcription factor 2; CTGF, connective tissue growth factor; DKK1, dickkopf1; ACVR2A, Activin A Receptor Type 2A; CTNNBIP1, Catenin Beta Interacting Protein 1; Pit-1, type III sodium-dependent Pi cotransporter-1; Mef2c, myocyte enhancer factor 2c; Rhob, ras homolog family member B; NFIA, nuclear factor IA.

## References

[1] Clarke B. (2008). Normal bone anatomy and physiology. Clin. J. Am. Soc. Nephrol..

[2] Alford A.I., Kozloff K.M., Hankenson K.D. (2015). Extracellular matrix networks in bone remodeling. Int. J. Biochem. Cell Biol..

[3] Feng X. (2009). Chemical and Biochemical Basis of Cell-Bone Matrix Interaction in Health and Disease. Curr. Chem. Biol..

[4] Zhou R., Guo Q., Xiao Y., Guo Q., Huang Y., Li C., Luo X. (2021). Endocrine role of bone in the regulation of energy metabolism. Bone Res..

[5] Shao J., Wang Z., Yang T., Ying H., Zhang Y., Liu S. (2015). Bone Regulates Glucose Metabolism as an Endocrine Organ through Osteocalcin. Int. J. Endocrinol..

[6] Du Y., Zhang L., Wang Z., Zhao X., Zou J. (2021). Endocrine Regulation of Extra-skeletal Organs by Bone-derived Secreted Protein and the effect of Mechanical Stimulation. Front. Cell Dev. Biol..

[7] Sun T., Yu X. (2022). FGF23 Actions in CKD-MBD and in Other Organs during CKD. Curr. Med. Chem..

[8] Hadjidakis D.J., Androulakis I.I. (2006). Bone remodeling. Ann. N. Y. Acad. Sci..

[9] Weaver C.M., Gordon C.M., Janz K.F., Kalkwarf H.J., Lappe J.M., Lewis R., Zemel B. (2016). The National Osteoporosis Foundation’s position statement on peak bone mass development and lifestyle factors: A systematic review and implementation recommendations. Osteoporos. Int..

[10] Bonjour J.-P., Chevalley T., Ferrari S., Rizzoli R. (2009). The importance and relevance of peak bone mass in the prevalence of osteoporosis. Salud Publica Mex..

[11] Levine M.A. (2012). Assessing bone health in children and adolescents. Indian. J. Endocrinol. Metab..

[12] Proia P., Amato A., Drid P., Korovljev D., Vasto S., Baldassano S. (2021). The Impact of Diet and Physical Activity on Bone Health in Children and Adolescents. Front. Endocrinol..

[13] Office of the Surgeon General (2004). Reports of the Surgeon General. Bone Health and Osteoporosis: A Report of the Surgeon General.

[14] Covic A., Vervloet M., Massy Z., Torres P.U., Goldsmith D., Brandenburg V., Mazzaferro S., Evenepoel P., Bover J., Apetrii M. (2018). Bone and mineral disorders in chronic kidney disease: Implications for cardiovascular health and ageing in the general population. Lancet Diabetes Endocrinol..

[15] Desbiens L.-C., Goupil R., Madore F., Mac-Way F. (2020). Incidence of fractures in middle-aged individuals with early chronic kidney disease: A population-based analysis of CARTaGENE. Nephrol. Dial. Transplant..

[16] Khairallah P., Nickolas T.L. (2020). The young, the uremic and the broken. Nephrol. Dial. Transplant..

[17] Vilaca T., Salam S., Schini M., Harnan S., Sutton A., Poku E., Allen I.E., Cummings S.R., Eastell R. (2020). Risks of Hip and Nonvertebral Fractures in Patients With CKD G3a-G5D: A Systematic Review and Meta-analysis. Am. J. Kidney Dis..

[18] Genant H.K., Cooper C., Poor G., Reid I., Ehrlich G., Kanis J., Nordin B.E.C., Barrett-Connor E., Black D., Bonjour J.-P. (1999). Interim report and recommendations of the World Health Organization Task-Force for Osteoporosis. Osteoporos. Int..

[19] Lewiecki E.M., Watts N.B. (2009). New guidelines for the prevention and treatment of osteoporosis. South. Med. J..

[20] Pisani P., Renna M.D., Conversano F., Casciaro E., Di Paola M., Quarta E., Muratore M., Casciaro S. (2016). Major osteoporotic fragility fractures: Risk factor updates and societal impact. World J. Orthop..

[21] Kuo T.R., Chen C.H. (2017). Bone biomarker for the clinical assessment of osteoporosis: Recent developments and future perspectives. Biomark. Res..

[22] Chan J.J., Cupples L.A., Kiel D.P., O’Donnell C.J., Hoffmann U., Samelson E.J. (2015). QCT Volumetric Bone Mineral Density and Vascular and Valvular Calcification: The Framingham Study. J. Bone Miner. Res..

[23] Kiel D.P., Kauppila L.I., Cupples L.A., Hannan M.T., O’Donnell C.J., Wilson P.W.F. (2001). Bone loss and the progression of abdominal aortic calcification over a 25 year period: The Framingham Heart Study. Calcif. Tissue Int..

[24] Leow K., Szulc P., Schousboe J.T., Kiel D.P., Teixeira-Pinto A., Shaikh H., Sawang M., Sim M., Bondonno N., Hodgson J.M. (2021). Prognostic Value of Abdominal Aortic Calcification: A Systematic Review and Meta-Analysis of Observational Studies. J. Am. Heart Assoc..

[25] Schulz E., Arfai K., Liu X., Sayre J., Gilsanz V. (2004). Aortic calcification and the risk of osteoporosis and fractures. J. Clin. Endocrinol. Metab..

[26] Naves M., García M.R., López J.B.D., Alonso C.G., Andía J.B.C. (2008). Progression of vascular calcifications is associated with greater bone loss and increased bone fractures. Osteoporos. Int..

[27] Coen G., Ballanti P., Mantella D., Manni M., Lippi B., Pierantozzi A., Di Giulio S., Pellegrino L., Romagnoli A., Simonetti G. (2009). Bone turnover, osteopenia and vascular calcifications in hemodialysis patients. A histomorphometric and multislice CT study. Am. J. Nephrol..

[28] Cascio V.L., Bertoldo F., Gambaro G., Gasperi E., Furlan F., Colapietro F., Cascio C.L., Campagnola M. (1999). Urinary galactosyl-hydroxylysine in postmenopausal osteoporotic women: A potential marker of bone fragility. J. Bone Miner. Res..

[29] Forsprecher J., Wang Z., Goldberg H.A., Kaartinen M.T. (2011). Transglutaminase-mediated oligomerization promotes osteoblast adhesive properties of osteopontin and bone sialoprotein. Cell Adh Migr..

[30] Holm E., Gleberzon J.S., Liao Y., Sørensen E.S., Beier F., Hunter G.K., Goldberg H.A. (2014). Osteopontin mediates mineralization and not osteogenic cell development in vitro. Biochem. J..

[31] Si J., Wang C., Zhang D., Wang B., Hou W., Zhou Y. (2020). Osteopontin in Bone Metabolism and Bone Diseases. Med. Sci. Monit..

[32] Cannata-Andia J.B., Roman-Garcia P., Hruska K. (2011). The connections between vascular calcification and bone health. Nephrol. Dial. Transplant..

[33] Boyce B.F., Xing L. (2007). Biology of RANK, RANKL, and osteoprotegerin. Arthritis Res. Ther..

[34] Luo J., Yang Z., Ma Y., Yue Z., Lin H., Qu G., Huang J., Dai W., Li C., Zheng C. (2016). LGR4 is a receptor for RANKL and negatively regulates osteoclast differentiation and bone resorption. Nat. Med..

[35] Takegahara N., Kim H., Choi Y. (2022). RANKL biology. Bone.

[36] Andreo-López M.C., Contreras-Bolívar V., García-Fontana B., García-Fontana C., Muñoz-Torres M. (2023). The Influence of the Mediterranean Dietary Pattern on Osteoporosis and Sarcopenia. Nutrients.

[37] Marini H., Bitto A., Altavilla D., Burnett B.P., Polito F., Di Stefano V., Minutoli L., Atteritano M., Levy R.M., D’Anna R. (2008). Breast safety and efficacy of genistein aglycone for postmenopausal bone loss: A follow-up study. J. Clin. Endocrinol. Metab..

[38] Carmen Naranjo M., Garcia I., Bermudez B., Lopez S., Cardelo M.P., Abia R., Muriana F.J.G., Montserrat-de la Paz S. (2016). Acute effects of dietary fatty acids on osteclastogenesis via RANKL/RANK/OPG system. Mol. Nutr. Food Res..

[39] Bucay N., Sarosi I., Dunstan C.R., Morony S., Tarpley J., Capparelli C., Scully S., Tan H.L., Xu W., Lacey D.L. (1998). Osteoprotegerin-deficient mice develop early onset osteoporosis and arterial calcification. Genes. Dev..

[40] Hofbauer L.C., Schoppet M. (2004). Clinical implications of the osteoprotegerin/RANKL/RANK system for bone and vascular diseases. JAMA.

[41] Carrillo-López N., Panizo S., Alonso-Montes C., Martínez-Arias L., Avello N., Sosa P., Dusso A.S., Cannata-Andía J.B., Naves-Díaz M. (2019). High-serum phosphate and parathyroid hormone distinctly regulate bone loss and vascular calcification in experimental chronic kidney disease. Nephrol. Dial. Transplant..

[42] Panizo S., Cardus A., Encinas M., Parisi E., Valcheva P., López-Ongil S., Coll B., Fernandez E., Valdivielso J.M. (2009). RANKL increases vascular smooth muscle cell calcification through a RANK-BMP4-dependent pathway. Circ. Res..

[43] Helas S., Goettsch C., Schoppet M., Zeitz U., Hempel U., Morawietz H., Hofbauer L.C. (2009). Inhibition of receptor activator of NF-kappaB ligand by denosumab attenuates vascular calcium deposition in mice. Am. J. Pathol..

[44] Samelson E.J., Miller P.D., Christiansen C., Daizadeh N.S., Grazette L., Anthony M.S., Egbuna O., Wang A., Siddhanti S.R., Cheung A.M. (2014). RANKL inhibition with denosumab does not influence 3-year progression of aortic calcification or incidence of adverse cardiovascular events in postmenopausal women with osteoporosis and high cardiovascular risk. J. Bone Miner. Res..

[45] Azizieh F.Y., Shehab D., Al Jarallah K., Gupta R., Raghupathy R. (2019). Circulatory Levels of RANKL, OPG, and Oxidative Stress Markers in Postmenopausal Women With Normal or Low Bone Mineral Density. Biomark. Insights.

[46] Xu S., Wang Y., Lu J., Xu J. (2012). Osteoprotegerin and RANKL in the pathogenesis of rheumatoid arthritis-induced osteoporosis. Rheumatol. Int..

[47] Fadda S., Hamdy A., Abulkhair E., Elsify H.M., Mostafa A. (2015). Serum levels of osteoprotegerin and RANKL in patients with rheumatoid arthritis and their relation to bone mineral density and disease activity. Egypt. Rheumatol..

[48] Nitta K., Akiba T., Uchida K., Otsubo S., Takei T., Yumura W., Kabaya T., Nihei H. (2004). Serum osteoprotegerin levels and the extent of vascular calcification in haemodialysis patients. Nephrol. Dial. Transplant..

[49] Osorio A., Ortega E., Torres J.M., Sanchez P., Ruiz-Requena E. (2013). Biochemical markers of vascular calcification in elderly hemodialysis patients. Mol. Cell Biochem..

[50] Mohammadpour A.H., Shamsara J., Nazemi S., Ghadirzadeh S., Shahsavand S., Ramezani M. (2012). Evaluation of RANKL/OPG Serum Concentration Ratio as a New Biomarker for Coronary Artery Calcification: A Pilot Study. Thrombosis.

[51] Li B., Yao Q., Guo S., Ma S., Dong Y., Xin H., Wang H., Liu L., Chang W., Zhang Y. (2019). Type 2 diabetes with hypertensive patients results in changes to features of adipocytokines: Leptin, Irisin, LGR4, and Sfrp5. Clin. Exp. Hypertens.

[52] Westendorf J.J., Kahler R.A., Schroeder T.M. (2004). Wnt signaling in osteoblasts and bone diseases. Gene.

[53] Pereira L.A.L., Meng C., Amoedo M.A.G., Pinto M.T.D.S.C., Mendes F., Marques M.A.M.P., Weigert A.L.L. (2023). Etelcalcetide controls secondary hyperparathyroidism and raises sclerostin levels in hemodialysis patients previously uncontrolled with cinacalcet. Nefrología.

[54] Kulkarni N., Halladay D., Miles R., Gilbert L., Frolik C., Galvin R., Martin T., Gillespie M., Onyia J. (2005). Effects of parathyroid hormone on Wnt signaling pathway in bone. J. Cell Biochem..

[55] Silva B.C., Bilezikian J.P. (2015). Parathyroid hormone: Anabolic and catabolic actions on the skeleton. Curr. Opin. Pharmacol..

[56] Mödder U.I., Hoey K.A., Amin S., McCready L.K., Achenbach S.J., Riggs B.L., Khosla S. (2011). Relation of Age, Gender, and Bone Mass to Circulating Sclerostin Levels in Women and Men. J. Bone Miner. Res. Off. J. Am. Soc. Bone Miner. Res..

[57] Asadipooya K., Abdalbary M., Ahmad Y., Kakani E., Monier-Faugere M.-C., El-Husseini A. (2021). Bone Quality in CKD Patients: Current Concepts and Future Directions—Part I. Kidney Dis..

[58] Gomes T.S., Aoike D.T., Baria F., Graciolli F.G., Moyses R.M., Cuppari L. (2017). Effect of Aerobic Exercise on Markers of Bone Metabolism of Overweight and Obese Patients with Chronic Kidney Disease. J. Ren. Nutr..

[59] Ardawi M.-S.M., Al-Kadi H., Rouzi A., Qari M.H. (2011). Determinants of serum sclerostin in healthy pre- and postmenopausal women. J. Bone Miner. Res..

[60] Peng J., Dong Z., Hui Z., Aifei W., Lianfu D., Youjia X. (2021). Bone Sclerostin and Dickkopf-related protein-1 are positively correlated with bone mineral density, bone microarchitecture, and bone strength in postmenopausal osteoporosis. BMC Musculoskelet. Disord..

[61] Kuo T.-H., Lin W.-H., Chao J.-Y., Wu A.-B., Tseng C.-C., Chang Y.-T., Liou H.-H., Wang M.-C. (2019). Serum sclerostin levels are positively related to bone mineral density in peritoneal dialysis patients: A cross-sectional study. BMC Nephrol..

[62] Gorter E.A., Reinders C.R., Krijnen P., Appelman-Dijkstra N.M., Schipper I.B. (2022). Serum sclerostin levels in osteoporotic fracture patients. Eur. J. Trauma. Emerg. Surg..

[63] Lu J.-W., Syu R.-J., Wang C.-H., Hsu B.-G., Tsai J.-P. (2022). Serum Sclerostin Level Is Negatively Associated with Bone Mineral Density in Hemodialysis Patients. Medicina.

[64] Jean G., Chazot C., Bresson E., Zaoui E., Cavalier E. (2016). High Serum Sclerostin Levels Are Associated with a Better Outcome in Haemodialysis Patients. Nephron.

[65] Ardawi M.-S.M., Rouzi A., Al-Sibiani S., Al-Senani N.S., Qari M.H., Mousa S. (2012). High serum sclerostin predicts the occurrence of osteoporotic fractures in postmenopausal women: The Center of Excellence for Osteoporosis Research Study. J. Bone Miner. Res..

[66] Pereira L., Magalhaes J., Mendonca L., Neto R., Santos J., Carvalho C.G., Oliveira A., Beco A., Frazao J. (2022). Evaluation of Renal Osteodystrophy and Serum Bone-Related Biomarkers in a Peritoneal Dialysis Population. J. Bone Miner. Res..

[67] Lv W., Guan L., Zhang Y., Yu S., Cao B., Ji Y. (2016). Sclerostin as a new key factor in vascular calcification in chronic kidney disease stages 3 and 4. Int. Urol. Nephrol..

[68] De Maré A., Opdebeeck B., Neven E., D’Haese P.C., Verhulst A. (2022). Sclerostin Protects Against Vascular Calcification Development in Mice. J. Bone Miner. Res..

[69] Fang Y., Ginsberg C., Seifert M., Agapova O., Sugatani T., Register T.C., Freedman B.I., Monier-Faugere M.-C., Malluche H., Hruska K.A. (2014). CKD-induced wingless/integration1 inhibitors and phosphorus cause the CKD-mineral and bone disorder. J. Am. Soc. Nephrol..

[70] Carrillo-López N., Martínez-Arias L., Fernández-Villabrille S., Ruiz-Torres M.P., Dusso A., Cannata-Andía J.B., European Renal Osteodystrophy (EUROD) Workgroup (2021). Role of the RANK/RANKL/OPG and Wnt/β-Catenin Systems in CKD Bone and Cardiovascular Disorders. Calcif. Tissue Int..

[71] Saag K.G., Petersen J., Brandi M.L., Karaplis A.C., Lorentzon M., Thomas T., Maddox J., Fan M., Meisner P.D., Grauer A. (2017). Romosozumab or Alendronate for Fracture Prevention in Women with Osteoporosis. N. Engl. J. Med..

[72] Hwang E.Y., Jeong M.S., Park E.-K., Kim J.H., Jang S.B. (2014). Structural characterization and interaction of periostin and bone morphogenetic protein for regulation of collagen cross-linking. Biochem. Biophys. Res. Commun..

[73] Bonnet N., Standley K.N., Bianchi E.N., Stadelmann V., Foti M., Conway S.J., Ferrari S.L. (2009). The matricellular protein periostin is required for sost inhibition and the anabolic response to mechanical loading and physical activity. J. Biol. Chem..

[74] Bonnet N., Conway S.J., Ferrari S.L. (2012). Regulation of beta catenin signaling and parathyroid hormone anabolic effects in bone by the matricellular protein periostin. Proc. Natl. Acad. Sci. USA.

[75] Gossiel F., Scott J.R., Paggiosi M., Naylor K., McCloskey E.V., Peel N.F., Walsh J.S., Eastell R. (2018). Effect of Teriparatide Treatment on Circulating Periostin and Its Relationship to Regulators of Bone Formation and BMD in Postmenopausal Women with Osteoporosis. J. Clin. Endocrinol. Metab..

[76] Khan R.A., Bhandari U., Kapur P., Jain A., Farah F. (2019). Effects of rosuvastatin (added to hypocaloric diet) on serum periostin, adiponectin, proinflammtory cytokines levels and hepatic steatosis in non-alcoholic fatty liver disease patients with dyslipidemia. Clin. Epidemiol. Glob. Health.

[77] Rousseau J.C., Sornay-Rendu E., Bertholon C., Chapurlat R., Garnero P. (2014). Serum periostin is associated with fracture risk in postmenopausal women: A 7-year prospective analysis of the OFELY study. J. Clin. Endocrinol. Metab..

[78] Kim B.-J., Rhee Y., Kim C.H., Baek K.H., Min Y.-K., Kim D.-Y., Ahn S.H., Kim H., Lee S.H., Lee S.-Y. (2015). Plasma periostin associates significantly with non-vertebral but not vertebral fractures in postmenopausal women: Clinical evidence for the different effects of periostin depending on the skeletal site. Bone.

[79] Anastasilakis A.D., Polyzos S.A., Makras P., Savvides M., Sakellariou G.T., Gkiomisi A., Papatheodorou A., Terpos E. (2014). Circulating periostin levels do not differ between postmenopausal women with normal and low bone mass and are not affected by zoledronic acid treatment. Horm. Metab. Res..

[80] Zhu Y., Ji J.-J., Wang X.-D., Sun X.-J., Li M., Wei Q., Ren L.-Q., Liu N.-F. (2021). Periostin promotes arterial calcification through PPARγ-related glucose metabolism reprogramming. Am. J. Physiol. Heart Circ. Physiol..

[81] Sun X.-J., Ma W.-Q., Zhu Y., Liu N.-F. (2021). POSTN promotes diabetic vascular calcification by interfering with autophagic flux. Cell Signal.

[82] Alesutan I., Henze L.A., Boehme B., Luong T.T.D., Zickler D., Pieske B., Eckardt K.-U., Pasch A., Voelkl J. (2022). Periostin Augments Vascular Smooth Muscle Cell Calcification via &beta;-Catenin Signaling. Biomolecules.

[83] Ryu J., Kim H.J., Chang E.-J., Huang H., Banno Y., Kim H.-H. (2006). Sphingosine 1-phosphate as a regulator of osteoclast differentiation and osteoclast-osteoblast coupling. EMBO J..

[84] Ishii M., Kikuta J., Shimazu Y., Meier-Schellersheim M., Germain R.N. (2010). Chemorepulsion by blood S1P regulates osteoclast precursor mobilization and bone remodeling in vivo. J. Exp. Med..

[85] Bae S.J., Lee S.H., Ahn S.H., Kim H.-M., Kim B.-J., Koh J.-M. (2016). The circulating sphingosine-1-phosphate level predicts incident fracture in postmenopausal women: A 3.5-year follow-up observation study. Osteoporos. Int..

[86] Ardawi M.-S.M., Rouzi A.A., Al-Senani N.S., Qari M.H., Elsamanoudy A.Z., Mousa S.A. (2018). High Plasma Sphingosine 1-phosphate Levels Predict Osteoporotic Fractures in Postmenopausal Women: The Center of Excellence for Osteoporosis Research Study. J. Bone Metab..

[87] Lee S.H., Lee S.Y., Lee Y.S., Kim B.J., Lim K.H., Cho E.H., Kim G.S. (2012). Higher circulating sphingosine 1-phosphate levels are associated with lower bone mineral density and higher bone resorption marker in humans. J. Clin. Endocrinol. Metab..

[88] Alam M.S., Getz M., Safeukui I., Yi S., Tamez P., Shin J., Haldar K. (2012). Genomic expression analyses reveal lysosomal, innate immunity proteins, as disease correlates in murine models of a lysosomal storage disorder. PLoS ONE.

[89] Morris T.G., Borland S.J., Clarke C.J., Wilson C., Hannun Y.A., Ohanian V., Canfield A.E., Ohanian J. (2018). Sphingosine 1-phosphate activation of ERM contributes to vascular calcification. J. Lipid Res..

[90] Fernández-Pisonero I., López J., Onecha E., Dueñas A.I., Maeso P., Crespo M.S., Román J.A.S., García-Rodríguez C. (2014). Synergy between sphingosine 1-phosphate and lipopolysaccharide signaling promotes an inflammatory, angiogenic and osteogenic response in human aortic valve interstitial cells. PLoS ONE.

[91] Goettsch C., Rauner M., Pacyna N., Hempel U., Bornstein S.R., Hofbauer L.C. (2011). miR-125b regulates calcification of vascular smooth muscle cells. Am. J. Pathol..

[92] Goettsch C., Hutcheson J.D., Aikawa E. (2013). MicroRNA in cardiovascular calcification: Focus on targets and extracellular vesicle delivery mechanisms. Circ. Res..

[93] Panizo S., Naves-Díaz M., Carrillo-López N., Martínez-Arias L., Fernández-Martín J.L., Ruiz-Torres M.P., Cannata-Andía J.B., Rodríguez I. (2016). MicroRNAs 29b, 133b, and 211 Regulate Vascular Smooth Muscle Calcification Mediated by High Phosphorus. J. Am. Soc. Nephrol..

[94] Eskildsen T., Taipaleenmäki H., Stenvang J., Abdallah B.M., Ditzel N., Nossent A.Y., Bak M., Kauppinen S., Kassem M. (2011). MicroRNA-138 regulates osteogenic differentiation of human stromal (mesenchymal) stem cells in vivo. Proc. Natl. Acad. Sci. USA.

[95] Inose H., Ochi H., Kimura A., Fujita K., Xu R., Sato S., Iwasaki M., Sunamura S., Takeuchi Y., Fukumoto S. (2009). A microRNA regulatory mechanism of osteoblast differentiation. Proc. Natl. Acad. Sci. USA.

[96] Hassan M.Q., Gordon J.A., Beloti M.M., Croce C.M., Wijnen AJ V., Stein J.L., Lian J.B. (2010). A network connecting Runx2, SATB2, and the miR-23a~27a~24-2 cluster regulates the osteoblast differentiation program. Proc. Natl. Acad. Sci. USA.

[97] Kahai S., Lee S.-C., Lee D.Y., Yang J., Li M., Wang C.-H., Jiang Z., Zhang Y., Peng C., Yang B.B. (2009). MicroRNA miR-378 regulates nephronectin expression modulating osteoblast differentiation by targeting GalNT-7. PLoS ONE.

[98] Zhang Y., Xie R.L., Croce C.M., Stein J.L., Lian J.B., Van Wijnen A.J., Stein G.S. (2011). A program of microRNAs controls osteogenic lineage progression by targeting transcription factor Runx2. Proc. Natl. Acad. Sci. USA.

[99] Li Z., Hassan M.Q., Jafferji M., Aqeilan R.I., Garzon R., Croce C.M., Lian J.B. (2009). Biological functions of miR-29b contribute to positive regulation of osteoblast differentiation. J. Biol. Chem..

[100] Kapinas K., Kessler C., Ricks T., Gronowicz G., Delany A.M. (2010). miR-29 modulates Wnt signaling in human osteoblasts through a positive feedback loop. J. Biol. Chem..

[101] Zhang J., Fu W., He M., Xie W., Lv Q., Wan G., Li G., Wang H., Lu G., Hu X. (2011). MiRNA-20a promotes osteogenic differentiation of human mesenchymal stem cells by co-regulating BMP signaling. RNA Biol..

[102] Sugatani T., Vacher J., Hruska K.A. (2011). A microRNA expression signature of osteoclastogenesis. Blood.

[103] Nakasa T., Shibuya H., Nagata Y., Niimoto T., Ochi M. (2011). The inhibitory effect of microRNA-146a expression on bone destruction in collagen-induced arthritis. Arthritis Rheum..

[104] Sugatani T., Hruska K.A. (2007). MicroRNA-223 is a key factor in osteoclast differentiation. J. Cell Biochem..

[105] Wang Y., Li L., Moore B.T., Peng X.-H., Fang X., Lappe J.M., Recker R.R., Xiao P. (2012). MiR-133a in human circulating monocytes: A potential biomarker associated with postmenopausal osteoporosis. PLoS ONE.

[106] Kelch S., Balmayor E.R., Seeliger C., Vester H., Kirschke J.S., van Griensven M. (2017). miRNAs in bone tissue correlate to bone mineral density and circulating miRNAs are gender independent in osteoporotic patients. Sci. Rep..

[107] Kocijan R., Muschitz C., Geiger E., Skalicky S., Baierl A., Dormann R., Plachel F., Feichtinger X., Heimel P., Fahrleitner-Pammer A. (2016). Circulating microRNA Signatures in Patients With Idiopathic and Postmenopausal Osteoporosis and Fragility Fractures. J. Clin. Endocrinol. Metab..

[108] Li H., Wang Z., Fu Q., Zhang J. (2014). Plasma miRNA levels correlate with sensitivity to bone mineral density in postmenopausal osteoporosis patients. Biomarkers.

[109] Meng J., Zhang D., Pan N., Sun N., Wang Q., Fan J., Zhou P., Zhu W., Jiang L. (2015). Identification of miR-194-5p as a potential biomarker for postmenopausal osteoporosis. PeerJ.

[110] Vimalraj S., Selvamurugan N. (2013). MicroRNAs: Synthesis, Gene Regulation and Osteoblast Differentiation. Curr. Issues Mol. Biol..

[111] Chao C.-T., Han D.-S., Huang J.-W. (2021). Circulating microRNA-125b Levels Are Associated With the Risk of Vascular Calcification in Healthy Community-Dwelling Older Adults. Front. Cardiovasc. Med..

[112] Chao C.-T., Liu Y.-P., Su S.-F., Yeh H.-Y., Chen H.-Y., Lee P.-J., Chen W.-J., Lee Y.-M., Huang J.-W., Chiang C.-K. (2017). Circulating MicroRNA-125b Predicts the Presence and Progression of Uremic Vascular Calcification. Arterioscler. Thromb. Vasc. Biol..

[113] Fernández-Villabrille S., Martín-Carro B., Martín-Vírgala J., Alonso-Montes C., Palomo-Antequera C., García-Castro R., López-Ongil S., Dusso A.S., Fernández-Martín J.L., Naves-Díaz M. (2023). MicroRNA-145 and microRNA-486 are potential serum biomarkers for vascular calcification. Nephrol. Dial. Transplant..

[114] Hackl M., Heilmeier U., Weilner S., Grillari J. (2016). Circulating microRNAs as novel biomarkers for bone diseases—Complex signatures for multifactorial diseases?. Mol. Cell. Endocrinol..

